# Prevalence, Molecular Profile and Antibiotic Resistance of *Listeria* Species in Retail Beef Products in North‐West Province, South Africa: A Cross‐Sectional Analysis

**DOI:** 10.1002/vms3.70680

**Published:** 2025-11-14

**Authors:** Nduduzo C. Mtshali, Nomakorinte Gcebe, Rebone Moerane, Abiodun A. Adesiyun

**Affiliations:** ^1^ Department of Production Animal Studies, Faculty of Veterinary Science University of Pretoria Pretoria South Africa; ^2^ Bacteriology Department, Onderstepoort Veterinary Research Agricultural Research Council Pretoria South Africa; ^3^ Department of Basic Veterinary Sciences, School of Veterinary Medicine, Faculty of Medical Sciences University of the West Indies St. Augustine Trinidad and Tobago

**Keywords:** antibiograms, beef and beef products, *Listeria*, North‐West Province, serogroup, South Africa, virulence factors

## Abstract

This cross‐sectional study determined the occurrence, distribution, molecular characteristics and antibiograms of *Listeria* species recovered from beef and beef products retailed in the North‐West Province, South Africa. The study also investigated the factors associated with the contamination of these products by *Listeria* spp. and their characteristics. Conventional methods and polymerase chain reaction (PCR) were employed to detect and characterize the isolates of *Listeria* spp. In contrast, the disc diffusion method was used to determine their susceptibility to 16 antimicrobial agents. Four hundred beef products were randomly collected from 30 retail outlets across the North‐West Province. The prevalence of *Listeria monocytogenes* and other *Listeria* spp. was 6% (24/400) and 30.5% (122/400), respectively (*p* < 0.001). Of the five variables (district, size of outlet, type of beef and beef products, product display temperature and types of presentation), only the type of beef and beef products had a statistically significant (*p* = 0.034) effect on the occurrence of *L. monocytogenes*. In contrast, none had any significant effect on other *Listeria* spp. Among the 24 isolates of *L. monocytogenes*, all five serogroups assayed were detected, with the predominant ones being IIb (45.8%), IVb (20.8%) and IIa (20.8%). All eight virulence genes assayed were detected, with *actA* (50%), *inlB* (45.8%) and *inlA* (41.7%) being the most frequently detected. All isolates of *L. monocytogenes* (*n* = 24) and other *Listeria* spp. (*n* = 122) were resistant to one or more of the 16 antimicrobial agents tested. For *L. monocytogenes* isolates, resistance was high to nalidixic acid (100%), enrofloxacin (41.7%) and cefoxitin (37.5%) but low to clindamycin (8.3%) and sulphamethoxazole‐trimethoprim (8.3%). The frequency of multi‐drug resistance (MDR) in the *L. monocytogenes* isolates was 95.8% (23/24). Our study reveals the risk of human listeriosis in consumers of beef and beef products contaminated by virulent and antimicrobial‐resistant serogroups of *L. monocytogenes* in the North‐West Province of South Africa.

## Graphical abstract

The study determined the occurrence, molecular characteristics and antimicrobial susceptibility of *Listeria monocytogenes* and other *Listeria* spp. recovered from beef and beef products in the North‐West Province of South Africa.

The occurrence of virulent and antimicrobial‐resistant serogroups of *L. monocytogenes* in beef and beef products poses a food safety risk.

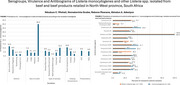

## 1 Introduction

Meat is a highly nutritious food, rich in proteins and vitamins essential for growth and other bodily functions. However, several factors, including biological, chemical and physical characteristics, can result in meat spoilage and threaten food safety (Ali and Alsayeqh 2022; González et al. 2020; Zhu et al. 2022). Meat and meat products have, therefore, been documented to pose a significant risk of consumer infections and outbreaks (Allam et al. 2018; González et al. 2020).

The pathogen *Listeria monocytogenes* significantly contributes to listeriosis, a foodborne zoonosis (Koopmans et al. 2023; Olanya et al. 2019). It has been responsible for numerous cases and outbreaks of human listeriosis (Allam et al. 2018; T. P. Liu et al. 2024; Speich et al. 2024). However, species of *Listeria* other than *L. monocytogenes*, such as *L. ivanovii* and *L. innocua* have also been reported to cause human and livestock listeriosis (Favaro et al. 2014; Gan et al. 2020; Koopmans et al. 2023; Liao et al. 2022).

Various food types, including meat and meat products, milk and milk products and vegetables, have been identified as carriers of *L. monocytogenes* and other *Listeria* spp., which can lead to human listeriosis (European Food Safety Authority and European Centre for Disease Prevention and Control 2018; McLauchlin et al. 2020). *L. monocytogenes* is a successful human pathogen due to its characteristics, including serogroups and virulence genes (Gana et al. 2024c, Gana et al. 2024b; Koopmans et al. 2023; Matle et al. 2020), which influence its frequency in listeriosis cases. Several serogroups of *L. monocytogenes*, including IIa, IIb, IIc and IVb, have been documented (Zhang et al. 2020). Several studies have reported varying frequencies of serogroups and virulence genes in *L. monocytogenes* isolated from foods (Koopmans et al. 2023; Matle et al. 2019; Terentjeva et al. 2021). However, some are more frequently involved in human cases of listeriosis, such as the *Listeria* Pathogenicity Island (LIPI)‐1 or the *prf*A gene cluster (*prfA*, *plcA*, *hly*, *mpl*, *actA* and *plcB*), which are involved in vacuolar escape (*hly* and *plcA*), actin‐based motility (*actA*) and cell‐cell spread (*mpl* and *plc*) (Wagner et al. 2022). The entry of *L. monocytogenes* into human host cells is mainly mediated by the prominent virulence factors, Internalin A (*InlA*) and *InlB* via receptor‐mediated endocytosis (Ireton et al. 2021). Furthermore, the LIPI‐3 virulence genes play a key role in the infectious life cycle and survival in the food processing environment (Koopmans et al. 2023; Wiktorczyk‐Kapischke et al. 2023). Therefore, the serogroups and presence of virulence genes carried by strains of *L. monocytogenes* significantly affect their pathogenicity and virulence (Bouymajane et al. 2021; Koopmans et al. 2023; Wiktorczyk‐Kapischke et al. 2023).

Medical and veterinary practitioners' indiscriminate use of antimicrobials in humans and animals has contributed to the increased emergence of antimicrobial resistance (Caneschi et al. 2023; Kasimanickam et al. 2021). The situation is exacerbated in developing countries, where, although policies on antimicrobial agents may exist, they are not frequently enforceable due to challenges in enforcement resulting from a lack of funding, personnel or laws that permit the over‐the‐counter purchase of antimicrobials (Van et al. 2020). There is a global increase in the prevalence of resistance to antimicrobial agents, particularly those considered priorities in treating listeriosis in humans and animals (Reis et al. 2022

South Africa experienced a massive outbreak of human listeriosis in 2018 (Allam et al. 2018), which was attributed to the consumption of a contaminated meat product (National Institute of Communicable Diseases 2018). Following the outbreak, several studies have been conducted, including determining the prevalence and molecular characteristics of *L. monocytogenes* strains in imported and local meat in the nine provinces of South Africa (Mafuna et al. 2021; Matle et al. 2019). Using PCR, genomic characterization of *L. monocytogenes* recovered from food processing environments (Mafuna et al. 2021); also, recently, the genomic characterization of *L. monocytogenes* and *L. innocua* isolated from the beef production chain and retail outlets (Gana et al. 2023, Gana et al. 2024c) and the prevalence of serogroups and virulence of *L. monocytogenes* strains that contaminated beef and beef products at retail outlets in Mpumalanga (Moabelo et al. 2023) and Gauteng (Gana et al. 2024b) provinces were documented.

In South Africa, the increased resistance to antimicrobial agents is particularly more pronounced in the livestock industry, where there is limited control of the use of antimicrobial agents, complicated by the legal use of over‐the‐counter drugs, thus leading to no veterinary oversight of their use (Mupfunya et al. 2021; Van et al. 2020). Reports exist on the resistance of *L. monocytogenes* recovered from local and imported meat and meat products in the nine provinces in the country (Matle et al. 2019), retailed beef and beef products in Gauteng Province (Gana et al. 2024a), Mpumalanga Province (Moabelo 2022), and the Eastern Cape Province (Kayode and Okoh 2022).

To date, information is lacking on the occurrence and characteristics (serogroups, virulence genes and antibiograms) of *Listeria* spp. in beef and beef products sold at retail outlets across the North‐West Province, even though residents experienced cases and deaths during the recent nationwide outbreak (National Institute of Communicable Diseases 2018). Therefore, the current study was conducted to determine the prevalence of *L. monocytogenes* and other *Listeria* spp. in beef and beef products retailed in the North‐West Province and to characterize the *L. monocytogenes* regarding their serogroups and virulence genes and the antibiograms of all *Listeria* spp. Finally, the study investigated the effects of variables (district, retail outlet size and beef and beef product types on the prevalence and characteristics of *L. monocytogenes* and other *Listeria* spp.

## 2 Materials and Methods

### 2.1 Study Design

A cross‐sectional study was conducted to determine the prevalence, molecular characteristics and factors associated with the contamination of beef and beef products retailed in the North‐West Province of South Africa, by *Listeria* spp.

### 2.2 Sample Size Determination

The study's sample size was estimated using the formula of Thrusfield (2007). For the study, a reported prevalence of 14.7% (Matle et al. 2019) and a precision value of 3.5% were used, resulting in an estimated sample size of 401. However, 400 samples of beef and beef‐based products were collected from 30 randomly selected retail outlets across the province.

### 2.3 Sources, Types of Beef and Beef Samples and Transportation to the Laboratory for Processing

The type and number of beef and beef products collected followed the criteria earlier published for a study conducted in Mpumalanga Province, South Africa (Moabelo et al. 2023). Beef and beef products were collected from four categories of retail outlets (chain, large, medium and small/butcheries) across the districts of North‐West Province. The samples included raw beef, boerewors, minced beef, cold beef (ready‐to‐eat, RTE) and ‘biltong’. The distribution of the types of samples collected is shown in Table 1. All samples collected from the retail outlets were stored in 4°C coolers, transported promptly to the ARC‐Onderstepoort Veterinary Institution (ARC‐OVI) Bacteriology laboratory within 12 h, and processed within 48 h of collection.

**TABLE 1:** Demographic distribution of the samples collected from retail outlets.

Variable	Level	No. of samples tested	Percent rate (%)	95% CI	*p* value
District	Brits and Brits Mall	123	30.8	8.5562 to 41.443	0.017
	Waterfall and Magalies	50	12.5		
	Rustenburg	143	35.7		
	Phokeng and Foro	84	21.0		
Size of outlet	Chain	177	44.3	−3.4554 to 53.5554	0.068
	Large	121	30.3		
	Medium	97	24.3		
	Small	5	1.3		
Type of sample	Brisket/raw beef	214	53.5	−3.86124 to 43.94212	0.080
	Boerewors/minced beef	59	14.3		
	Beef patties/beef burger	21	5.3		
	Cold beef	69	17.3		
	Biltong	39	9.8		
Product display temperature	Room temperature	37	9.3	−68.1437 to 134.8103	0.293
	Chilled	322	80.5		
	Frozen	41	10.2		
Presentation at outlet	RTE	108	27	−242.2427 to 342.2427	0.275
	Raw	292	73		

Abbreviation: RTE, Ready to eat.

#### 2.3.1 Isolation and Identification of *Listeria* spp

To isolate and identify *Listeria* spp., standard bacteriological and polymerase chain reactions (PCR) were used to qualitatively analyse the samples, as earlier described (Matle et al. 2019; Moabelo et al. 2023).

#### 2.3.2 Enrichment of Samples

All beef (raw and RTE) samples were aseptically cut into small pieces using forceps and scissors, and 10 g of each was weighed on a weighing balance. Each 10 g sample was transferred aseptically into a stomacher bag containing 90 mL of ONE Broth‐*Listeria* enrichment broth (LEB) (ThermoFisher Scientific, SA). The inoculated LEB was then incubated aerobically for 48 h at 35°C.

#### 2.3.3 Isolation of *Listeria* spp

A loopful of inoculated LEB was streaked for isolation on Brilliance *Listeria* agar (BLA) plates, followed by 48 h incubation at 35°C. Suspected characteristic colonies exhibited by other *Listeria* spp. (blue colonies without a halo) and *L. monocytogenes* (blue colonies with a white halo), as described by Jamali et al. (2013), were subcultured on BLA to obtain pure cultures.

### 2.4 Molecular Identification of *Listeria* Isolates

#### 2.4.1 Extraction of DNA From Enriched Broth Cultures

The study used the boiling–centrifugation method to extract DNA from the broth cultures, as described by Soumet et al. (1994). The extracted DNA was then used to characterize further the *L. monocytogenes* isolates using PCR.

#### 2.4.2 PCR Screening of Broth Cultures for *Listeria* spp

Conventional PCR assays targeting the *prs* gene were used to determine the presence of the genus *Listeria* in the broth cultures (LEB) of all samples, as Doumith et al. (2004) described. The details of the PCR preparation used in the current study were earlier documented (Moabelo et al. 2023). The same cPCR assay method was used to characterize *L. monocytogenes* regarding its serogroups.

#### 2.4.3 DNA Extraction From Isolates of *Listeria* spp

We extracted DNA from pure cultures of individual isolates of *L. monocytogenes* using the method described by Soumet et al. (1994). The extracted DNA was used or PCR to screen for serogroup and virulence gene profiling, as documented in the literature. The extracted DNA was transferred into sterile Eppendorf tubes and stored at −20°C.

#### 2.4.4 Characterization of *L. monocytogenes* Isolates

##### 2.4.4.1 Multiplex PCR Assay to Detect *L. monocytogenes* Serogroups

Multiplex PCR (mPCR) that targets five fragments of *L. monocytogenes*, namely, *imo1118, imo0737, orf2110, orf2819* and *prs*, was used to determine the serogroups of *L. monocytogenes* as described by Doumith et al. (2004). The primer sequences used for the PCR to detect the serogroups of *L. monocytogenes* are shown in Table S1. *L. monocytogenes* ATCC 19111 was used as a positive control. The products were subjected to electrophoresis in 3% agarose gel, and a gel documentation system (Vacutec, All SA) was used to capture the images. A sample of the PCR gel images used to detect the serogroups of *L. monocytogenes* is shown in Figure S1.

##### 2.4.4.2 Detection of Virulence Genes in *L. monocytogenes* Isolates

The presence of eight selected virulence genes in *L. monocytogenes* isolates was determined, as described by Rawool et al. (2007). mPCR was used to detect eight virulence genes (*plcA*, *hlyA*, *actA, inlB*, *iap*, *inlA*, *inlC* and *inlJ*) in *L. monocytogenes* isolates in two reactions (Table S2).

*Reaction 1* (mPCR 1) contained five pairs (reverse and forward) of primers (plcA, hlyA, actA, inIB and iap), while *Reaction 2 (mPCR 2)* consisted of three pairs of primers (inIA, inIC and inIJ). The DNA template preparation from the *L. monocytogenes* isolates, PCR assay and agarose gel electrophoresis of the PCR products were performed as described by Rawool et al. (2007). A sample of the PCR gel images used to detect the virulence genes in the isolates of *L. monocytogenes* is shown in Figure S2.

### 2.5 Determination of the Susceptibility of *L. monocytogenes* and Other *Listeria* spp. To Antimicrobial Agents

The susceptibility of all isolates of *Listeria* spp. recovered from the retail outlet sources was phenotypically determined against 16 antimicrobial agents (ThermoFisher Scientific, South Africa). These antimicrobial agents are readily available to livestock farmers and used by veterinarians and medical doctors in the country to treat infections caused by *L. monocytogenes* and other bacterial pathogens. The Kirby–Bauer disk diffusion method was used according to the guidelines and interpretation of the Clinical and Laboratory Standards Institute (Clinical and Laboratory Standards Institute, 2018). For the study, *L. monocytogenes* ATCC 19111, *Listeria innocua* ATCC 33090 and *Campylobacter fetus* ATCC 273737373 were used as controls. The inhibition zones were determined as susceptible (S), intermediate (I) or resistant (R) to the antimicrobial agents tested. For antimicrobial agents for which the cut‐off values for susceptibility were not stated for *Listeria*, the values provided for staphylococci were used as recommended by Conter et al. (2009).

### 2.6 Data Analysis

The Statistical Package for Social Sciences (SPSS) and Epi Info were used to generate percentages or prevalence of *Listeria* spp. according to the districts of retail outlets and their size, sample types, product display temperatures at the point of sale and product presentation at outlets. The data obtained in the current study on the prevalence of *Listeria* spp. as they relate to the variables investigated and the characteristics (serogroups, virulence and antibiograms) of *Listeria* spp. were entered into Microsoft Excel 2016 and analysed.

The data were then analysed using the statistical software R and STATA 15, and the association of the variables was determined using Fisher's Exact and chi‐square tests. The significance level was set at an alpha level of 0.05, and the percentages were calculated at a 95% confidence interval.

## 3 Results

### 3.1 Overall Prevalence of *L. monocytogenes* and Other *Listeria* spp. in Retail Outlets

Of the 400 beef and beef product samples collected from 30 retail outlets, 6% (24/400) tested positive for *L. monocytogenes* and 30.5% (122/400) for other *Listeria* spp. The difference was statistically significant (*p* < 0.001).

### 3.2 Prevalence of *L. monocytogenes* Isolated From Retail Outlets According to the Associated Factors

**FIGURE 1:**
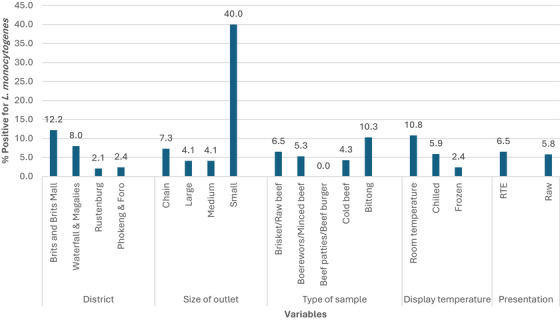
Prevalence of *L. monocytogenes* in beef and beef products according to the variables investigated.

Figure 1 shows the prevalence of *L. monocytogenes* according to the five variables investigated. The prevalence of *L. monocytogenes* varied considerably but with no statistically significant effect on four of the five variables as follows: ranging from 2.1% (3/143) in Rustenburg) to 12.2% (15/123) in Brits and Brits Mall (*p* = 0.084) by the region, from 4.1% (5/121, 4/97) in large and medium outlets, respectively to 40% (2/5) in small abattoirs (*p* = 0.211) according to the size of the outlets, from 2.4% (1/41) in samples kept at frozen temperature to 10.8% (4/37) in those kept at room temperature, according to the display temperature of the product at sale (*p* = 0.121), and from 5.8% (17/292) in raw beef to 6.5% (7/108) in RTE by the presentation at sale (*p* = 0.805). However, the type of beef and beef products had a statistically significant effect (*p* = 0.034) on the prevalence of *L. monocytogenes*, ranging from 0% (0/21) in beef patties and beef burgers to 10.3% (4/39) in ‘biltong’.

### 3.3 Prevalence of Other *Listeria* spp. Isolated from Retail Outlets According to the Associated Factors

**FIGURE 2:**
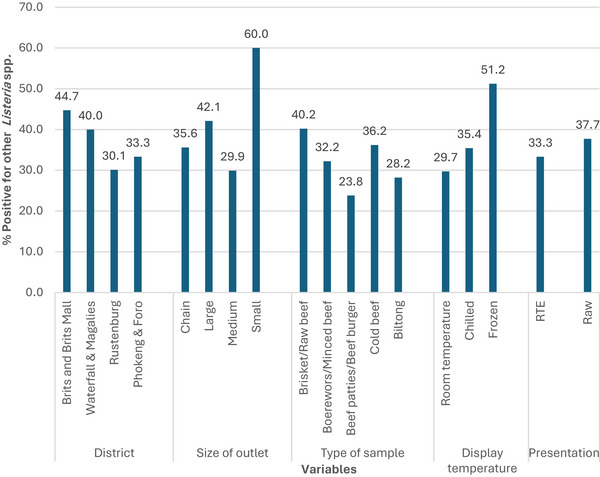
Prevalence of other *Listeria* spp. in beef and beef products according to the variables investigated.

According to the five variables investigated, none of the factors was significantly associated with the occurrence of other *Listeria* spp. in beef and beef products (Figure 2). By the district, the lowest prevalence of *Listeria* spp. was found in Rustenburg, 30.1% (43/143), and the highest in Brits and Brits Mall, 44.7% (55/123) (*p* = 0.080). Within the sizes of the retail outlets, the prevalence ranged from 29.9% (29/97) in medium outlets to 60% (3/5) in small outlets (*p* = 0.192). According to the types of beef and beef products, the lowest prevalence of other *Listeria* spp. was detected in beef patties and beef burgers, 23.8% (5/21), and the highest in brisket and raw beef, 40.2% (86/214) (*p* = 0.390). Regarding the temperature of the beef and beef products at the point of sale, the prevalence of other *Listeria* spp. was lowest in the products at room temperature, 29.7% (11/37) and highest in frozen products, 51.2% (21/41) (*p* = 0.0540), while for the presentation of products, the prevalence of other *Listeria* spp. was 33.3% (36/108) and 37.7% (110/292) in RTE and raw beef respectively. The difference was not statistically significant (*p* = 0.424).

### 3.4 Comparison of the Prevalence of *L. monocytogenes* and Other *Listeria* spp. According to the Associated Factors

In four of the five variables investigated for their effect on the detection of *Listeria* spp. and *L. monocytogenes* in beef and beef products, the highest prevalence detected was statistically significantly higher for *Listeria* spp., than for *L. monocytogenes*. Specifically, for *Listeria* spp. and *L. monocytogenes*, the respective highest prevalence according to the variables was as follows: the region (44.7% vs. 12.2%, *p* < 0.001), type of sample (40.2% vs. 10.3%, *p* < 0.0001), product display temperature at sale (51.2% vs. 10.8%, *p* < 0.0001) and presentation at the point of sale (37.7% vs. 6.5%, *p* < 0.001). However, for the size of the retail outlet, the highest prevalence for the other *Listeria* spp. was (60%, 3/5) and for *L. monocytogenes* (40%, 2/5), but the differences were not statistically significantly different (*p* = 0.527).

### 3.5 Serogroups of *L. monocytogenes* Detected in the Study

Overall, the five serogroups assayed in the study were detected by PCR. The frequency distribution of the serogroups assayed in the 24 isolates of *L. monocytogenes* was as follows: 4.2% (1/24) for IVb‐1; 8.3% (2/24) for IIc (1/2c‐3c); 20.8% (5/24) for IIa (1/2a‐3a); 20.8% (5/24) for IVb (4a‐4b, 4d‐4e) and 45.8% (11/24) for IIb (1/2b‐3b). The differences were statistically significant (*p* = 0.003). A total of 10 serotypes 2a, 2b, 2c, 3a, 3b, 3c, 4a, 4b, 4d and 4e) were detected in the 24 isolates of *L. monocytogenes* (Table 2).

**TABLE 2:** Frequency of serogroups of *L. monocytogenes* isolates recovered from beef and beef products according to the region, size of outlet, and sample type.

		No. (%) of isolates positive for:				
District	No. of isolates tested^a^	1/2a‐3a (IIa)	1/2b‐3b (IIb)	1/2c ‐3c (IIc)	4b‐4d‐4e (IVb)	IVb‐1
Brits and Brits Mall	15	1 (6.7)	8 (53.3)	2 (13.3)	3 (20.0)	1 (6.7)
Waterfall and Magalies	4	1 (25.0)	2 (50.0)	0 (0.0)	1 (25.0)	0 (0.0)
Rustenburg	3	1 (3.3)	1 (33.3)	0 (0.0)	1 (33.3)	0 (0.0)
Phokeng and Foro	2	2 (100.0)	0 (0.0)	0 (0.0)	0 (0.0)	0 (0.0)
*p* value		0.136	0.020	1	0.021	1
Total	24	5 (20.8)	11 (45.8)	2 (8.3)	5 (20.8)	1(4.2)

		No. (%) of isolates positive for:				
Size of outlets	No. of isolates tested^a^	1/2a‐3a (IIa)	1/2b‐3b (IIb)	1/2c ‐3c (IIc)	4b‐4d‐4e (IVb)	IVb‐1
Chain	13	3 (23.1)	5 (38.5)	1 (7.7)	3 (23.1)	1 (7.7)
Large	5	1 (20.0)	3 (60.0)	0 (0.0)	1 (20.0)	0 (0.0)
Medium	4	1 (25.0)	2 (50.0)	0 (0.0)	1 (25.0)	0 (0.0)
Small	2	0 (0.0)	1 (50.0)	1 (50.0)	0 (0.0)	0 (0.0)
*p* value		0.983	0.866	0.315	0.983	1
Total	24	5 (20.8)	11 (45.8)	2 (8.3)	5 (20.8)	1(4.2)

		No. (%) of isolates positive for:				
Product type	No. of isolates tested^a^	1/2a‐3a (IIa)	1/2b‐3b (IIb)	1/2c ‐3c (IIc)	4b‐4d‐4e (IVb)	IVb‐1
Brisket/raw beef	14	2 (14.3)	7 (50.0)	1 (7.1)	3 (21.4)	1 (7.1)
Boerewors/minced	3	1 (33.3)	1 (33.3)	0 (0.0)	1 (33.3)	0 (0.0)
Cold beef	3	1 (33.3)	1 (33.3)	0 (0.0)	1 (33.3)	0 (0.0)
Biltong	4	1 (25.0)	2 (50.0)	1 (25.0)	0 (0.0)	0 (0.0)
*p* value		0.807	0.918	0.324	0.072	1
Total	24	5 (20.8)	11 (45.8)	2 (8.3)	5 (20.8)	1 (4.2)

^a^ No *L. monocytogenes* isolate was recovered from beef patties and burgers.

The district locations of the retail outlet only had a statistically significant effect on the prevalence of serogroups of *L. monocytogenes* for serogroups 1/2b‐3b (IIb) (*p* = 0.020) and 4b‐4d‐4c (*p* = 0.021). For the other three serogroups, the differences in the frequency of detection were not statistically significant (*p* > 0.05) (Table 2).

The distribution of serogroups among *L. monocytogenes* varied according to the size of the retail outlets, but no significant effect was detected (Table 2). The isolates from the chain supermarkets were positive for all five serogroups. In contrast, three [1/2a‐3a (IIa), 1/2b‐3b (IIb), 4b‐4d‐4e (IVb)] were detected in isolates from large and medium supermarkets. Only two serogroups [1/2b‐3b (IIb) and 1/2c‐3c (IIc)] were found in isolates recovered from small supermarkets.

All five serogroups of *L. monocytogenes* assayed were detected in each of the beef and products. However, the type of product had no statistically significant effect on the prevalence of the serogroups directed (*p* > 0.05) (Table 2).

### 3.6 Frequency of Virulence Genes in *L. monocytogenes* Isolates According to the District, Size of Retail Outlet and Type of Beef and Beef Products

Although the district locations of the retail outlets did not statistically significantly (*p* > 0.05) affect the frequency of detection of the virulence genes in the *L. monocytogenes* isolates, their distribution was variable (Table 3). The virulence genes detected at the highest frequency per region were as follows: *actA* (73.3%) in Brits and Mall; *iap* (100%), *inlB* (100%) and *inlC* (100%) in Waterfall and Magalies; *Iap* (100%), *inlB* (100%) and *inlC* (100%) in Rustenburg; and only *actA* (50%) and *inlB* (50%) were detected in Phokeng and Foro district (Table 3).

**TABLE 3:** Frequency of detection of virulence factors in *L. monocytogenes* according to the region, size of retail outlets and sample type.

		No. (%) of isolates positive for:							
District	No. of isolates tested	*act A*	*iap*	*inl A*	*inl B*	*inl C*	*inl J*	*hly A*	*plc A*
Brits and Brits Mall	15	11 (73.3)	1 (6.7)	7 (46.7)	3 (20.0)	2 (13.3)	0 (0.0)	8 (53.3)	0 (0.0)
Waterfall and Magalies	4	0 (0.0)	4 (100)	1 (25.0)	4 (100.0)	4 (100.0)	1 (25.0)	0 (0.0)	2 (50.0)
Rustenburg	3	0 (0.0)	3 (100)	2 (66.7)	3 (100.0)	3 (100.0)	1 (33.3)	0 (0.0)	1 (33.3)
Phokeng and Foro	2	1 (50.0)	0 (0.0)	0 (0.0)	1 (50.0)	0 (0.0)	0 (0.0)	0 (0.0)	0 (0.0)
*p* value		0.198	0.161	0.095	0.348	0.144	0.188	0.391	0.194

		No. (%) of isolates positive for:							
Retail size	No. of isolates tested	*act A*	*iap*	*inl A*	*inl B*	*inl C*	*inl J*	*hly A*	*plc A*
Chain	13	7 (53.8)	5 (38.5)	4 (30.8)	6 (46.2)	5 (38.5)	1 (2.4)	4 (30.8)	1 (2.4)
Large	5	2 (40.0)	2 (40.0)	3 (60.0)	2 (40.0)	3 (60.0)	1(20.0)	1 (20.0)	1 (20.0)
Medium	4	2 (50.0)	1 (25.0)	3 (75.0)	3 (75.0)	1 (25.0)	0 (0.0)	2 (50.0)	1 (25.0)
									
Small	2	1 (50.0)	0 (0.0)	0 (0.0)	0 (0.0)	0 (0.0)	0 (0.0)	1 (50.0)	0 (0.0)
*p* value		0.964	0.068	0.088	0.179	0.091	0.331	0.756	0.154

	No. of	No. (%) of isolates positive for:							
Sample Type	isolates tested	*act A*	*iap*	*inl A*	*inl B*	*inl C*	*inl J*	*hly A*	*plc A*
Raw beef	14	8 (57.1)	4 (28.6)	6 (42.9)	6 (42.9)	4 (28.6)	1 (7.1)	4 (28.6)	1 (7.1)
Boerewors/minced beef	3	0 (0.0)	3 (−100)	2 (66.7)	3 (100.0)	3 (100)	1 (33.3)	0 (0.0)	2 (66.7)
Cold beef	3	2 (66.7)	0 (0.0)	2 (66.7)	1 (33.3)	1 (33.3)	0 (0.0)	2 (66.7)	0 (0.0)
Biltong	4	2 (50)	1 (25)	0 (0.0)	1 (25)	1 (25)	0 (0.0)	2 (33.3)	0 (0.0)
*p* value		0.077	0.198	0.068	0.076	0.098	0.292	0.1	0.337

The size of the retail outlet did not significantly (*p* > 0.05) affect the frequency of virulence genes, as shown in Table 3. All eight virulence genes were detected in the isolates of *L. monocytogenes* recovered from beef and beef products collected from the chain and large supermarkets, while in large supermarkets (seven genes), and small outlets (only two genes) were detected. For chain supermarkets, the frequency range of virulence genes was from 2.4% (i*nlJ* and *plcA*) to 53.8% (*actA*) (*p* = 0.0108), while in large supermarkets, the frequency ranged from 20% *(hlyA* and *plcA*) to 60% (*inlA* and *inlC*) (*p* = 0.303). In isolates from fr medium‐sized supermarkets, the lowest frequency (0%) was detected for the *inlJ* gene and the highest frequency was for *inlA* (75%) (*p* = 0.1429). However, for small supermarkets, the two genes detected were *actA* and *hlyA*, each detected at a frequency of 50%. Similarly, the types of beef and beef products did not have a statistically significant (*p* > 0.05) effect on the frequency of virulence genes detected (Table 3). For the isolates of *L. monocytogenes* recovered from raw beef, the eight genes assayed were detected, ranging in prevalence from 7.1% (*inlJ*) to 57.1% (*actA*), and the difference was statistically significant (*p* = 0.0046). Of the isolates from boerewors/minced beef, six of the eight virulence genes were detected, with the prevalence being lowest (0%) for *actA* and *hlyA* genes, but highest (100%) for *iap* and *inlB* genes (*p* > 0.05). Five virulence genes were detected in the isolates of *L. monocytogenes* from cold beef. The prevalence ranged from 0% (*iap, inlJ* and *plcA*) to 66.7% (*actA, inlA and hltA*) (*p* = 0.40), and for ‘biltong’ isolates, five genes were detected, with the lowest prevalence being 0.0% (*inlA* and *plcA*) and the highest being 33.3% (*hlyA*) (*p* = 0.455).

### 3.7 Antimicrobial Resistance Profiles Exhibited by *Listeria* spp. Isolates

**FIGURE 3:**
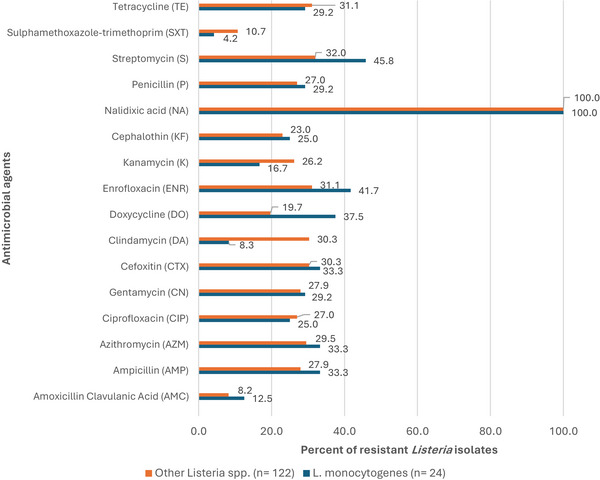
Prevalence of resistance to antimicrobial agents in *L. monocytogenes* and *Listeria* spp. isolates recovered from beef and beef products.

All 146 *Listeria* isolates (24 *L. monocytogenes* and 122 other *Listeria* spp.) exhibited resistance to more than one of the 16 antimicrobial agents assayed. The prevalence of resistance ranged from 4.2% (sulphamethoxazole‐trimethoprim, SXT) to 100% (nalidixic acid) (Figure 3). Overall, the prevalence of resistance was low (< 10%) to two antimicrobial agents (amoxicillin clavulanic acid and sulphamethoxazole‐trimethoprim). Moderate resistance (< 10 to < 30%) was to eight antimicrobial agents (doxycycline, cephalothin, kanamycin, clindamycin, ciprofloxacin, penicillin, gentamycin and ampicillin), and comparatively high (> 30%–100%) resistance to six antimicrobials (azithromycin, cefoxitin, tetracycline, enrofloxacin, streptomycin and nalidixic acid). The differences in resistance to the 16 antimicrobials were statistically significant (*p* < 0.001).

Among the 24 isolates of *L. monocytogenes*, the prevalence of resistance was high to nalidixic acid (100%), streptomycin (45.8%), and enrofloxacin (41.7%) but low to sulphamethoxazole‐trimethoprim (4.2%) and clindamycin (8.3%). The differences were statistically significant (*p* < 0.001).

For the 122 isolates of other *Listeria* spp., resistance was high to nalidixic acid. (100%), streptomycin (32%) and tetracycline (31.1%), but comparatively low for amoxicillin‐clavulanic Acid (8.2%) and sulphamethoxazole‐trimethoprim (10.7%). The differences were statistically significant (*p* < 0.001).

The prevalence of resistance to the antimicrobial agents by *L. monocytogenes* and other *Listeria* spp. was statistically significantly different (*p* = 0.026; 8.3% vs. 30.3%) for only clindamycin.

For the antimicrobials used as treatment options for human listeriosis, the prevalence of resistance in *L. monocytogenes* was as follows: penicillin (29.2%), ampicillin (33.3%), gentamycin (29.2%), sulphamethoxazole‐trimethoprim (4.2%) and tetracycline (29.2%).

#### 3.7.1 Antimicrobial Resistance Profiles Exhibited by *L. monocytogenes* and Other *Listeria* spp. According to the District

Table 4 shows the frequency of resistance to antimicrobial agents for the 24 isolates of *L. monocytogenes* from beef and beef products, according to the district location of the retail outlets. For the 16 antimicrobial agents tested, the district location of the retail outlet had a statistically significant (*p* = 0.025) effect on cephalothin only. However, for the 122 isolates of other *Listeria* spp., the district had a statistically significant impact on the frequency of resistance to 13 antimicrobials but not to three, streptomycin (*p* = 0.336), nalidixic acid (*p* = 1) and sulphamethoxazole‐trimethoprim (*p *= 0.843).

**TABLE 4:** Antimicrobial resistance profile of *L. monocytogenes* and other *Listeria* spp. by district.

		No. (%) of isolates resistant to:															
District	No of *L. monocytogenes* isolates tested	P	AMC	AMP	KF	CTX	S	CN	K	TE	DO	NA	CIP	ENR	DA	SXT	AZM
Brits and Brit Mall	15	3 (20.0)	3 (20.0)	6 (40.0)	2 (13.3)	7 (46.7)	6 (40.0)	3 (20.0)	5 (33.3)	4 (26.7)	5 (33.3)	15 (100.0)	2 (13.3)	7 (46.7)	2 (13.3)	2 (13.3)	4 (26.7)
Waterfall	4	2 (50.0)	0 (0.0)	1 (25.0)	2 (50.0)	0 (0.0)	1 (25.0)	1 (25.0)	0 (0.0)	1 (25.0)	2 (50.0)	4 (100.0)	2 (50.0)	1 (25.0)	0 (0.0)	0 (0.0)	1 (25.0)
Rustenburg	3	1 (33.3)	0 (0.0)	0 (0.0)	1 (33.3	2 (66.7)	1 (33.3)	1 (33.3)	0 (0.0)	1 (33.3)	0 (0.0)	3 (100.0)	1 (33.3	1 (33.3	0 (0.0)	0 (0.0)	2 (66.7)
Phokeng	2	0 (0.0)	0 (0.0)	1 (50.0)	1 (50.0)	0 (0.0)	2 (100.0)	1 (50.0)	0 (0.0)	1 (50.0)	1 (50.0)	2 (100.0)	1 (50.0)	1 (50.0)	0 (0.0)	0 (0.0)	1 (50.0)
*p* value		0.092	0.391	0.077	0.025	0.191	0.852	0.807	0.391	0.914	0.066	1	0.362	0.86	0.391	1	0.537

		No. (%) of isolates resistant to:															
District		P	AMC	AMP	KF	CTX	S	CN	K	TE	DO	NA	CIP	ENR	DA	SXT	AZM
Brits and Brit Mall	40	14 (35.0)	5 (12.5)	15 (37.5)	9 (22.5)	11 (27.5)	10 (25.0)	9 (22.5)	9 (22.5)	11 (27.5)	8 (20.0)	40 (100.0)	8 (20.0)	10 (25.0)	14 (35.0)	5 (12.5)	11 (27.5)
Waterfall	16	4 (25.0)	1 (6.3)	3 (18.8)	5 (31.3)	5 (31.3)	8 (50.0)	6 (37.5)	4 (25.0)	6 (37.5)	3 (18.8)	16 (100.0)	5 (31.3)	6 (37.5)	5 (31.3)	2 (12.5)	4 (25.0)
Rustenburg	40	10 (25.0)	2 (5.0)	10 (25.0)	8 (20.0)	11 (27.5)	14 (35.0)	14 (35.0)	12 (30.0)	13 (32.5)	7 (17.5)	40 (100.0)	12 (30.0)	14 (35.0)	7 (17.5)	3 (7.5)	13 (32.5)
Phokeng	26	6 (23.1)	2 (7.7)	9 (34.6)	6 (23.1)	9 (34.6)	8 (30.8)	6 (23.1)	6 (23.1)	8 (30.8)	7 (26.9)	26 (100.0)	8 (30.8)	8 (30.8)	9 (34.6)	2 (7.7)	8 (30.8)
*p* value		0.0012	0.017	0.007	0.002	0.0004	0.336	0.005	0.001	0.001	0.002	1	0.002	0.001	0.006	0.843	0.0004

Abbreviations: AMC, amoxicillin clavulanic acid; AMP, ampicillin; AZM, azithromycin; CIP, ciprofloxacin; CN, gentamycin; CTX, cefoxitin; DA, clindamycin; DO, doxycycline; ENR, enrofloxacin; K, kanamycin; KF, cephalothins; NA, nalidixic acid; P, penicillin; S, streptomycin; SXT, sulphamethozaxole‐trimethoprim; TE, tetracycline.

#### 3.7.2 Antimicrobial Resistance Profiles Exhibited by *L. monocytogenes* and Other *Listeria* spp. According to the Size of the Outlets

Table 5 shows the resistance distribution to the 16 antimicrobial agents according to the size of the retail outlets from which the beef and beef products were collected. Except for nalidixic acid, all the isolates of *L. monocytogenes* (*n* = 24) and other *Listeria* spp. (*n* = 122) were resistant. The frequency of resistance varied considerably within the four categories (chain, large, medium and small); however, the differences were not statistically significant (*p* > 0.05).

**TABLE 5:** Antimicrobial profiles of *L. monocytogenes* and *Listeria* spp. from beef and beef products by size of outlet.

		No. (%) of isolates resistant to:															
Retail size	No of *L. monocytogenes* isolates tested	P	AMC	AMP	KF	CTX	S	CN	K	TE	DO	NA	CIP	ENR	DA	SXT	AZM
Chain	13	3 (23.1)	1 (7.7)	1 (7.7)	3 (23.1)	6 (46.2)	7 (53.8)	2 (15.4)	2 (15.4)	4 (30.8)	5 (38.5)	13 (100.0)	4 (30.8)	5 (38.5)	2 (15.4)	0 (0.0)	5 (38.5)
Large	5	1 (20.0)	1 (20.0)	3 (60.0)	0 (0.0)	2 (40.0)	3 (60.0)	3 (60.0)	0 (0.0)	1 (20.0)	2 (40.0)	5 (100.0)	2 (40.0)	2 (40.0)	0 (0.0)	0 (0.0)	0 (0.0)
Medium	4	1 (25.0)	1 (25.0)	1 (25.0)	2 (50.0)	0 (0.0)	1 (25.0)	1 (25.0)	1 (25.0)	2 (50.0)	1 (25.0)	4 (100.0)	0 (0.0)	2 (50.0)	0 (0.0)	2 (50.0)	3 (75.0)
Small	2	1 (50.0)	0 (0.0)	1 (50.0)	0 (0.0)	1 (50.0)	0 (0.0)	0 (0.0)	2 (100.0)	0 (0.0)	0 (0.0)	2 (100.0)	0 (0.0)	0 (0.0)	0 (0.0)	0 (0.0)	0 (0.0)
*p* value		0.0259	0.1039	0.0575	0.222	0.0599	0.5346	0.143	0.2126	0.0947	0.0681	1	0.187	0.0616	0.391	1	0.213

		No. (%) of isolates resistant to:															
Retail size	No. of L*isteria* spp. isolates tested	P	AMC	AMP	KF	CTX	S	CN	K	TE	DO	NA	CIP	ENR	DA	SXT	AZM
Chain	50	15 (30.0)	6 (12.0)	19 (38.0)	10 (20.0)	11 (22.0)	19 (38.0)	14 (28.0)	7 (14.0)	15 (30.0)	11 (22.0)	50 (100.0)	11 (22.0)	16 (32.0)	16 (32.0)	6 (12.0)	15 (30.0)
Large	46	14 (30.4)	2 (4.3)	9 (19.6)	11 (23.9)	15 (32.6)	12 (26.1)	14 (30.4)	15 (32.6)	14 (30.4)	10 (21.7)	46 (100.0)	10 (21.7)	11 (23.9)	14 (30.4)	4 (8.7)	14 (30.4)
Medium	25	4 (16.0)	2 (8.0)	8 (32.0)	8 (32.0)	10 (40.0)	7 (28.0)	4 (16.0)	9 (36.0)	9 (36.0)	3 (12.0)	25 (100.0)	9 (36.0)	12 (48.0)	6 (24.0)	2 (8.0)	10 (40.0)
Small	1	1 (100.0)	0 (0.0)	0 (0.0)	0 (0.0)	0 (0.0)	1 (100.0)	1 (100.0)	0 (0.0)	0 (0.0)	0 (0.0)	1 (100.0)	1 (100.0)	0 (0.0)	1 (100.0)	0 (0.0)	0 (0.0)
*p* value		0.1022	0.0985	0.0757	0.0684	0.0728	0.5196	0.1062	0.0913	0.0597	0.0749	1	0.0952	0.0615	0.0799	0.2905	0.0629

Abbreviations: AMC: amoxicillin clavulanic acid, AMP: ampicillin, AZM: azithromycin, CIP: ciprofloxacin, CN: gentamycin, CTX: cefoxitin, DA: clindamycin, DO: doxycycline, ENR: enrofloxacin, K: kanamycin, KF: cephalothins, NA: nalidixic acid, P: penicillin, S: streptomycin, SXT: sulphamethozaxole‐trimethoprim, TE: tetracycline.

#### 3.7.3 Antimicrobial Resistance Profiles Exhibited by *L. monocytogenes* and Other *Listeria* spp. According to the Types of Beef and Beef Products

The occurrence of resistance exhibited by the isolates of *L. monocytogenes* and other *Listeria* spp. in the five beef and beef products (raw beef, boerewors, beef burgers, cold beef and ‘biltong’) from which they were recovered is shown in Table 6. Among the *L. monocytogenes* isolates, the type of beef and beef products had a significant effect on the frequency of resistance to three antimicrobials, namely, ampicillin (*p* = 0.0209), ciprofloxacin (*p* = 0.0288) and enrofloxacin (*p* = 0.0273). The frequency of resistance varied significantly (*p* < 0.05) among the other *Listeria* spp. isolated from the beef and beef products for 13 antimicrobials but not for three: streptomycin (*p* = 0.158), nalidixic acid (*p* = 1) and sulphamethoxazole‐trimethoprim (*p* = 0.6235).

**TABLE 6:** Antimicrobial profiles of *L. monocytogenes* and *Listeria* spp. from beef and beef products by type of products.

	No. of *L. monocytogenes*	No. (%) of isolates of *L. monocytogenes* resistant to:															
Type of sample^a^	isolates tested	P	AMC	AMP	KF	CTX	S	CN	K	TE	DO	NA	CIP	ENR	DA	SXT	AZM
Raw beef	14	3 (21.4)	1 (7.1)	3 (21.4)	6 (42.9)	5 (37.5)	7 (50.0)	1 (7.1)	2 (14.3)	5 (37.5)	6 (42.9)	14 (100.0)	2 (14.3)	5 (37.5)	2 (14.3)	1 (7.1)	6 (42.9)
Boerewors	3	1 (33.3)	0 (0.0)	1 (33.3)	0 (0.0)	2 (66.7)	1 (33.3)	1 (33.3)	0 (0.0)	1 (33.3)	1 (33.3)	3 (100.0)	1 (33.3)	1 (33.3)	0 (0.0)	0 (0.0)	1 (33.3)
Cold beef	3	0 (0.0)	2 (66.7)	1 (33.3)	0 (0.0)	0 (0.0)	2 (66.7)	2 (66.7)	0 (0.0)	1 (33.3)	1 (33.3)	3 (100.0)	1 (33.3)	2 (66.7)	0 (0.0)	1 (33.3)	1 (33.3)
Biltong	4	2 (50.0)	0 (0.0)	1 (25.0)	0 (0.0)	1 (25.0)	1 (25.0)	1 (25.0)	3 (75.0)	0 (0.0)	0 (0.0)	4 (100.0)	1 (25.0)	2 (50.0)	0 (0.0)	0 (0.0)	0 (0.0)
*p−value*		0.0966	0.322	0.0209	0.374	0.1082	0.680	0.0871	0.287	0.0712	0.074	1	0.0288	0.0273	0.3739	1	0.0741

		No. (%) of isolates of *L. monocytogenes* resistant to:															
Type of sample	No. of *Listeria* spp. isolates tested	P	AMC	AMP	KF	CTX	S	CN	K	TE	DO	NA	CIP	ENR	DA	SXT	AZM
Raw beef	72	19 (26.4)	4 (5.6)	22 (30.6)	15 (20.8)	21 (29.2)	27 (37.5)	21 (29.2)	19 (26.4)	17 (23.6)	14 (19.4)	72 (100.0)	20 (27.8)	20 (27.8)	21 (29.2)	8 (11.1)	22 (30.6)
Boerewors	16	5 (31.3)	2 (12.5)	2 (12.5)	4 (25.0)	5 (31.3)	4 (25.0)	2 (12.5)	3 (18.8)	8 (50.0)	1 (6.3)	16 (100.0)	5 (31.3)	4 (25.0)	2 (12.5)	2 (12.5)	3 (18.8)
Beef burgers	5	3 (60.0)	1 (20.0)	0 (0.0)	2 (40.0)	2 (40.0)	3 (60.0)	1 (20.0)	1 (20.0)	1 (20.0)	1 (20.0)	5 (100.0)	0 (0.0)	2 (40.0)	2 (40.0)	0 (0.0)	2 (40.0)
Cold beef	22	4 (18.2)	3 (13.6)	9 (40.9)	1 (4.5)	8 (36.4)	3 (13.6)	9 (40.9)	6 (27.3)	10 (45.5)	6 (27.3)	22 (100.0)	8 (36.4)	7 (31.8)	7 (31.8)	3 (13.6)	7 (31.8)
Biltong	7	3 (42.9)	1 (14.3)	2 (28.6)	3 (42.9)	1 (14.3)	2 (28.6)	3 (42.9)	2 (28.6)	2 (28.6)	3 (42.9)	7 (100.0)	1 (14.3)	2 (28.6)	5 (71.4)	0 (0.0)	2 (28.6)
*p* value		0.008	0.005	0.0358	0.019	0.0024	0.158	0.0077	0.0003	0.005	0.018	1	0.0292	0.0003	0.0188	0.6235	0.0009

Abbreviations: AMC, amoxicillin clavulanic acid; AMP, ampicillin; AZM, azithromycin; CIP, ciprofloxacin; CN, gentamycin; CTX, cefoxitin; DA, clindamycin; DO, doxycycline; ENR, enrofloxacin; K, kanamycin; KF, cephalothins; NA, nalidixic acid; P, penicillin; S, streptomycin; SXT, sulphamethozaxole‐trimethoprim; TE, tetracycline.

#### 3.7.4 Antimicrobial Resistance Patterns Exhibited by *L. monocytogenes* Isolates

Each of the 24 isolates of *L. monocytogenes* exhibited different resistance patterns (Table 7). The number of antimicrobial agents involved in the patterns ranged from 4 to 8, 4 to 5, 5 to 7 and 3 to 6 for isolates recovered from brisket/raw beef, boerewors/beef, cold beef and ‘biltong’, respectively. Twenty‐three (95.8%) of the 24 isolates exhibited multi‐drug resistance (MDR).

**TABLE 7:** Antimicrobial resistance patterns exhibited by *L. monocytogenes* isolates.

Type of sample isolated from	No. of Isolates	Resistance pattern	No. of antimicrobials
Brisket/raw beef (*n* = 14)	1 1 1 1 1 1 1 1 1 1 1 1 1 1	AMP‐CTX‐S‐K‐NA‐CIP‐DA‐ AZM KF‐S‐TE‐DO‐NA‐ENR KF‐DO‐NA‐ENR CTX‐S‐TE‐NA AMC‐AMP‐S‐DO‐NA‐AZM P‐AMP‐CTX‐NA‐ENR CTX‐S‐NA‐DA AMP‐K‐NA‐STX CTX‐DO‐NA‐ENR‐AZM AMP‐CTX‐TE‐NA P‐KF‐CN‐TE‐DO‐NA‐CIP P‐KF‐NA‐AZM KF‐S‐NA‐CIP‐AZM KF‐S‐TE‐DO‐NA‐ENR‐AZM	8 6 4 4 6 5 4 4 5 4 7 4 5 7
Boerewors/minced beef (*n* = 3)	1 1 1	AMP‐S‐DO‐NA‐CIP P‐CTX‐CN‐NA‐ENR CTX‐TE‐NA‐AZM	5 5 4
Cold beef (*n* = 3)	1 1 1	AMC‐CN‐TE‐NA‐ENR‐STX‐ AZM AMC‐S‐CN‐DO‐NA‐ENR AMP‐S‐CN‐NA‐CIP	7 6 5
Biltong (*n* = 4)	1 1 1 1	P‐CTX‐K‐NA P‐CN‐K‐NA‐CIP‐ENR AMP‐K‐NA S‐NA‐ENR	4 6 3 3

## 4 Discussion

Our study demonstrated that beef and beef products, including ready‐to‐eat (RTE) products, were positive for *L. monocytogenes*, which were carriers of pathogenic serogroups, virulence genes and exhibited resistance to several antimicrobial agents, which can pose a food safety and therapeutic risk to consumers. The food safety risk posed to consumers of beef‐based RTE products in South Africa is supported by the documentation that the RTE meat product ‘polony’ was implicated in the largest human listeriosis outbreak in South Africa, which resulted in 1060 cases and 216 deaths during 2017–2018 (Allam et al. 2018; Kaptchouang Tchatchouang et al. 2020; National Institute of Communicable Diseases 2018). In addition, the outbreak raised significant concern among the population and resulted in substantial economic losses (Olanya et al. 2019).

Following the outbreak, researchers investigated the risk of listeriosis associated with consuming beef and beef products and characterized the isolates of *L. monocytogenes* and *Listeria* spp. (Gana et al. 2024b; Keet and Rip 2021; Moabelo et al. 2023). These findings suggest that meat and meat products, particularly ready‐to‐eat (RTE) products, continue to pose a risk of listeriosis to human consumers. The differences in the prevalence of *Listeria*‐contamination of meat and meat products across the country can be attributed to the types of products tested (meat vs. beef), sample sources (retail outlets vs. abattoirs, meat processing plants and butcheries), animal sources (cattle vs. poultry, sheep, pigs and game) and geographical sources (local: one province vs. nine provinces vs. international). These factors influence the frequency of *L. monocytogenes* contamination in meat and meat products (Y. Liu et al. 2020; Meza‐Bone et al. 2023).

In our study, *Listeria* spp., other than *L. monocytogenes*, were detected at statistically significantly different prevalences of 30.5% and 6%, respectively, in beef and beef products from all sources. It is well established that *L. monocytogenes* is the most important human and animal pathogen among *Listeria* spp. (Kaptchouang Tchatchouang et al. 2020; T. P. Liu et al. 2024; Speich et al. 2024). Other *Listeria* spp., not speciated in the current study, such as *L. ivanovii*, are also known human and ruminant pathogens (Koopmans et al. 2023; Rossi et al. 2022), while *L. innocua* has been documented to cause listeriosis in immunocompromized humans (Favaro et al. 2014; Liao et al. 2022). The presence of other *Listeria* spp. as contaminants in beef products represents a potential food safety risk that warrants further investigation. Similarly, a comparatively higher prevalence of other *Listeria* spp., such as *L. innocua*, has been documented in meat and meat products by others (Gana et al. 2024b; Moabelo et al. 2023) than in *L. monocytogenes*.

Notably, of the five variables investigated, only the beef and beef product types had a statistically significant impact on the prevalence of *L. monocytogenes*. Our study detected the lowest prevalence (0%) of *L. monocytogenes* in beef patties and burgers and the highest in ‘biltong’ (10.3%), a local popular delicacy indigenous to South Africa. This finding is important because ‘biltong’ (both moist and dry) is a ready‐to‐eat (RTE) product widely consumed in the country, and *L. monocytogenes* has been documented at levels ranging from 3.6% to 9.7% in the product by others in the country (Gana et al. 2024b; Moabelo et al. 2023). The potential for contamination of ‘biltong’ during preparation and solar or controlled drying has been reported to contribute to the microbial quality of the product (Gavai et al. 2022; C. E. Karolenko et al. 2020, C. Karolenko et al. 2023).

Contrary to the findings, where only the beef and beef product type significantly affected the prevalence of *L. monocytogenes*, in a survey conducted in Gauteng Province, South Africa, Gana et al. (2024b) reported that the contamination of beef and beef products with *L. monocytogenes* was significantly affected by three variables (regional location, beef and beef product type of products and temperature of the product at the point of sale). On the other hand, in Mpumalanga Province, only the regional location of the retail outlets significantly affected the frequency of *L. monocytogenes* in beef and beef products (Moabelo et al. 2023). Matle et al. (2019) also reported that the occurrence of *L. monocytogenes* varied considerably according to the source of the samples. The differences in the occurrence of *L. monocytogenes* according to the five variables investigated, detected in five studies conducted in South Africa, may be multifactorial, including the sources of the samples tested, level of infection in cattle, contamination of beef at abattoirs and retail outlets, among others.

The prevalence of 6.5% detected for *L. monocytogenes* in brisket/raw beef in this study is lower than the 15.1% reported earlier for raw meat in the North‐West Province by Matle et al. (2019). The difference could be partly accounted for because the current study sampled only beef and beef products, whereas their research processed meat samples from various animal species (chicken, sheep, pigs, game and cattle), originating from local and international sources.

In our study, 5.3% of minced beef was contaminated by *L. monocytogenes*, which is higher than the 0.0% and 1% reported for ground beef and minced beef reported by others (Turanoglu et al. 2024; Uludağ et al. 2023) but considerably lower than the 0‐59% reported for ground beef in Brazil (Cavalcanti et al. 2022). The implication is that consuming minced beef from different countries poses potentially variable risks of human listeriosis, which may be attributable to the level of contamination of beef and beef products and the mincing process itself.

It is of food safety importance that all the beef patty and beef burger samples were negative (0%) for L. monocytogenes, indicating that the risk of listeriosis posed to consumers of these products is minimal in the North‐West Province of South Africa. A higher risk of human exposure to *L. monocytogenes* has been documented in beef products in Russia (13.2% Yushina et al. 2022) and Brazil (2.5%–59.4%, Reis et al. 2024). Differences in the contamination of beef and beef products and the degree of contamination during product preparation may have contributed to the discrepancy.

It is relevant that the five serogroups of *L. monocytogenes* we examined in the current study were detected in the 24 *L. monocytogenes* isolates, albeit at varying frequencies. Of potential etiological significance is that some of the serogroups and serotypes recovered in our study have been frequently detected in *L. monocytogenes* recovered from foods and cases of human listeriosis (Brown et al. 2023; Capita et al. 2019; Lachtara et al. 2021). The detection of several pathogenic serogroups at relatively high frequency in our beef and beef products, including RTE products, is therefore significant as it poses a food safety risk to consumers of contaminated products. The predominant serogroups detected in our study (IIb, IIa and IVb) were similar to those found in 33 *L. monocytogenes* isolates (IIa and IVb) from Mpumalanga Province (Moabelo et al. 2023) and in Gauteng Province (Gana et al. 2024b). It is, therefore, evident that across the three provinces, serotypes 2a, 3a, 1/2b, 3b, 4b, 4d, and 4e were the most common among the *L. monocytogenes* isolates that contaminated beef and beef products. Unlike our study, Matle et al. (2019) reported, in a survey of local and imported meat and meat products in the country, that the predominant serogroups were IIc. IIa, and IVb. In other countries, as found in our research, serogroup IIa (1/2a‐3a) was reported to be the most common serogroup of *L. monocytogenes* isolated from imported beef in Jordan (Obaidat 2020), in meat and meat products in China (Chen et al. 2019), and raw meat in Turkey (Arslan, and Baytur 2019).

Our finding of all eight virulence genes at high frequency in our beef and beef products, regardless of their sources, also poses food safety concerns to their consumers. This is relevant because virulence genes detected in our study have been demonstrated to play a significant role in the pathogenesis of human listeriosis (Bouymajane et al. 2021; Koopmans et al. 2023; Wiktorczyk‐Kapischke et al. 2023). The range of frequencies of the eight virulence genes assayed in *L. monocytogenes* from beef and beef products is the same (50%–100%) for the current study and as reported for isolates from Gauteng Province (50%–100%) (Gana et al. 2024b), but slightly higher than found in Mpumalanga Province, ranging from 21.2% to 100% (Moabelo et al. 2023). Matle et al. (2019) similarly detected virulence genes in *L. monocytogenes* from meat and meat products across nine provinces in the country and imported products, with a range of 20.3% to 98.7%.

In our study, the five variables investigated did not significantly affect the frequency of virulence genes in *L. monocytogenes*, consistent with findings in Gauteng Province (Gana et al. 2024b). However, Moabelo et al. (2023) reported that the size of retail outlets significantly affected the frequency of *InlB*. The differences in the carriage of virulence genes in *L. monocytogenes* recovered from sources in South Africa may reflect variations in the types of products and animal sources, among other factors.

Our finding of potential therapeutic significance is that all our 146 isolates of *Listeria* (including 24 *L. monocytogenes*) exhibited resistance to one or more antimicrobial agents. It is essential to note that, although the prevalence of resistance to antimicrobial agents varied, it was higher for *L. monocytogenes* (ten antimicrobials) than for other *Listeria* spp. (5 antimicrobials). Similar high occurrences of resistance to antimicrobial agents by *L. monocytogenes* recovered from foods and the environment have been reported elsewhere in the country (Gana et al. 2024a; Kayode et al. 2021; Matle et al. 2019), as well as in other countries (Boukili et al. 2020; Noll et al. 2018; Şanlibaba et al. 2020). Notably, each of the 24 *L. monocytogenes* isolates exhibited different resistance patterns, with 95.8% being MDR strains. The therapeutic implications posed by the risk of complications if MDR *L. monocytogenes* enter the human food chain are important. Similarly, a high prevalence of MDR *L. monocytogenes* (51.5%–100%) has been reported in South Africa by others (Gana et al. 2024a; Kayode and Okoh 2023; Matle et al. 2019; Moabelo et al. 2023) and elsewhere (Obaidat 2020; Şanlibaba et al. 2020). The widespread occurrence of MDR *L. monocytogenes* strains in the country has been suggested to reflect the selective pressure exerted by drug over‐prescription in clinical settings. Their heavy use as growth promoters and in therapy for farm animals may have resulted in the increased prevalence of bacterial resistance (Van et al. 2020).

In the country, some antimicrobial agents, such as tetracycline, are legally permitted for over‐the‐counter sale (Mupfunya et al. 2021; Van et al. 2020) and, therefore, are inexpensive; their use is not subject to veterinary supervision. This lack of oversight may contribute to the prevalence of resistance to tetracycline (29.2%) by *L. monocytogenes* isolates in the current study. These frequencies differ from those reported by others, such as 21.6%, 37.2%, 54.5% and 75%, which were earlier documented in meat isolates from the country (Gana et al. 2024a; Kayode and Okoh 2023; Matle et al. 2019; Moabelo 2022). The implications of this lack of veterinary oversight on the use of tetracycline in the country are concerning and should motivate the government to consider the potential for increased resistance to antimicrobials with therapeutic implications, as recommended in Sub‐Saharan African countries (Belachew et al. 2021). The therapeutic significance of detecting a relatively high prevalence of resistance to listeriosis cannot be overstated. Our findings suggest that the prevalence of resistance to antimicrobial agents ranged from 29.2% to 33.3% for ampicillin, gentamycin, penicillin and tetracycline, but only 4.2% for sulphamethoxazole‐trimethoprim, which is used in the treatment of human listeriosis (Reis et al. 2022; Ma et al. 2023). Based on our findings, sulphamethoxazole‐trimethoprim appears to be the best option for treating infections by *L. monocytogenes* in South Africa.

The occurrence of resistance to the 16 antimicrobials exhibited by other *Listeria* spp. at a comparatively similar frequency to *L. monocytogenes*, it can also not be ignored. Although they are considered non‐pathogenic, except for *L. ivanovii*, they have been implicated in listeriosis in immunocompromised humans (Favaro et al. 2014; Koopmans et al. 2023; Liao et al. 2022). Of therapeutic importance are these non‐pathogenic MDR *Listeria* spp. occur in the same food niche as pathogenic *L. monocytogenes*, the potential for transferring resistance genes and other genetic materials exists through several mechanisms (Goh et al. 2024; Li et al. 2022). This, therefore, poses a therapeutic concern, considering the high frequency of MDR in *L. monocytogenes* and other *Listeria* spp. in our study.

The geographical locations of the retail outlets significantly affected the prevalence of resistance to only cephalothin by *L. monocytogenes* in our study. In contrast, the location of the outlet significantly affected the prevalence of resistance to four antimicrobials (cefotaxime, cephalothin, streptomycin and penicillin by *L. monocytogenes* isolates recovered from beef and beef products in Gauteng Province (Gana et al. 2024a), while in Mpumalanga Province, significant effect on the prevalence of resistance was found to two antimicrobial agents (gentamycin and azithromycin) (Moabelo 2022). In contrast, for other *Listeria* spp., also recovered from beef and beef products, the regional location of the retail outlets significantly affected the prevalence of resistance to antimicrobial agents to 13 antimicrobial agents in our study, to eight in Gauteng Province (Gana et al. 2024a) and only two in Mpumalanga Province (Moabelo 2022). The disparity in the effects of geographical location on the prevalence of resistance to antimicrobial agents in the country has also been documented by others elsewhere (Anwar et al. 2022; Bouymajane et al. 2021).

Our findings revealed that the geographical location of retail outlets affected the prevalence of resistance to antimicrobial agents, with significant effects varying according to the size of the retail outlets. This is because a significant effect was found in only penicillin in the current study, among four antimicrobial agents (cefotaxime, cephalothin, clindamycin and penicillin) among *L. monocytogenes* in Gauteng Province (Gana et al. 2024a), and in none of the 12 antimicrobial agents in Mpumalanga Province (Moabelo 2022). Similar patterns of effect on the size of retail outlets were detected in the prevalence of resistance to antimicrobial agents among *Listeria* spp. isolated from the three provinces.

The type of beef and beef products significantly impacted the prevalence of resistance to three antimicrobials (ampicillin, ciprofloxacin and enrofloxacin) in the current study. The prevalence of resistance varied considerably among the other *Listeria* spp. for as many as 13 antimicrobials except for three (streptomycin, nalidixic acid and sulphamethoxazole‐trimethoprim). The fact that similar statistically significant effects of the type of beef and beef products on the prevalence of resistance exhibited by *L. monocytogenes* and other *Listeria* spp. in other provinces in the country (Gana et al. 2024b; Moabelo 2022) is an indication that the type of beef products consumed may pose a risk of exposure to antimicrobial‐resistant *L. monocytogenes*, with potential therapeutic implications in the province.

It is pertinent to mention that the disparity detected in the statistically significant effects of the retail outlets’ geographical location and size as well as the type of beef and beef products sampled in North‐West Province compared to the findings in similar studies in Gauteng and Mpumalanga provinces may be due in part to factors including the distribution of the four classes of retail outlets that receive beef and beef products from different sources, the frequency or overuse of antimicrobial agents on livestock farms, and the magnitude of cross‐contamination during slaughter or processing and at retail outlets. All these factors can potentially contribute to the significant association of the types of beef and beef products with the prevalence of resistance exhibited by *L. monocytogenes* and other *Listeria* spp. in our study.

Some limitations of the study included our isolation of only 24 (6%) *L. monocytogenes* from 400 beef and beef products are significantly lower than the 122 isolates of other *Listeria* species. Among all *Listeria* species, *L. monocytogenes* is recognized as the most significant cause of human listeriosis. The limited number of *L. monocytogenes* isolates recovered from the processed samples may influence the frequency of serogroups and virulence genes detected. Another limitation is that our study was conducted in three of the four municipal districts in the North‐West Province, which may impact the representativeness of our samples across the province. Finally, since the cut‐off values for susceptibility in the disc diffusion method for specific antimicrobial agents were not specified for *Listeria* species; we instead used those that Conter et al. (2009) recommended for staphylococci. Therefore, the susceptibility to specific antimicrobial agents was estimated based on zone sizes for *Listeria*.

## 5 Conclusions

The detection of *L. monocytogenes* (6%) strains, which belong to pathogenic serogroups and carry virulence genes in beef and beef products, pose significant concerns for food safety and public health for consumers. Equally important was our finding that all our isolates of *L. monocytogenes* and *Listeria* spp. exhibited resistance to one or more of the 16 antimicrobial agents, including antimicrobial agents used as options for treating human listeriosis, which could lead to therapeutic failure. Finally, the detection of pathogenic serogroups, virulent and antimicrobial‐resistant strains of *L. monocytogenes* in beef and beef products, including ready‐to‐eat (RTE) products, suggests that following the outbreak of human listeriosis in 2017–2018, the risk of human listeriosis in the North‐West Province remains.

## Author Contributions

**Nduduzo C. Mtshali**: data curation, formal analysis, investigation methodology, validation, visualization, writing – original draft, writing – review and editing. **Nomakorinte Gcebe**: conceptualization, funding acquisition, investigation methodology, project administration, supervision, validation, visualization, writing – review and editing. **Rebone Moerane**: conceptualization, funding acquisition, project administration, supervision, writing – review and editing. **Abiodun A. Adesiyun**: conceptualization, data curation, formal analysis, funding acquisition, investigation methodology, project administration, supervision, writing – original draft, writing – review and editing.

## Funding

The Red Meat Research and Development South Africa (RMRD‐SA) funded the project, which was awarded on 1 January 2019

## Ethics Statement

Before commencing the study, we received ethical approval from the following committees: the Research Ethics Committee (REC) (*REC 138‐19*) and the Animal Ethics Committee (AEC) (REC 138‐19) of the Faculty of Veterinary Science, University of Pretoria, South Africa, and Section 20 from the Department of Agriculture, Forestry and Fisheries (DAFF), [Number:12/11/1/1/8(1131)] South Africa.

## Consent

The researcher informed the managers and supervisors of the retail outlets before samples were collected at the selected retail outlets.

## Conflicts of Interest

The authors declare no conflicts of interest.

## Peer Review

The peer review history for this article is available at https://doi.org/10.1002/vms3.70680.

## Supporting information




**TABLE S1**: Primers used for mPCR serogrouping in this study (Doumith et al., 2004). Supporting **FIGURE S1**: PCR gel images obtained on 3% agarose gels to detect the serogroups of L. monocytogenes. **TABLE S2**: Primers used for mPCR virulence profiling (Rawool et al., 2017). **FIGURE S2**: PCR gel images obtained on 3% agarose gels to detect virulence genes in L. monocytogenes isolates.

## Data Availability

The data supporting this study's findings are available at upspace@up.ac.za and http://repository.up.ac.za, reference number 4870.
