# Supplementary material for: Prevalence, Molecular Profile and Antibiotic Resistance of Listeria Species in Retail Beef Products in North‐West Province, South Africa: A Cross‐Sectional Analysis
Source: Vet Med Sci. 2025 Nov 14;11(6):e70680. doi: 10.1002/vms3.70680 (PMC12617351; doi:10.1002/vms3.70680)
Supplement: Supplementary file 1 — TABLE S1: Primers used for mPCR serogrouping in this study (Doumith et al., 2004). Supporting FIGURE S1: PCR gel images obtained on 3% agarose gels to detect the serogroups of L. monocytogenes. TABLE S2: Primers used for mPCR virulence profiling (Rawool et al., 2017). FIGURE S2: PCR gel images obtained on 3% agarose gels to detect virulence genes in L. monocytogenes isolates. [file VMS3-11-e70680-s001.docx]

### **Supplementary Tables and Figures**

### **TABLE S1** Primers used for mPCR serogrouping in this study (Doumith *et al* 2004)

| **Assay** | **Primer** | **Primer sequence (5’ 3’)** | **Product sizes (bp)** |
| --- | --- | --- | --- |
| mPCR1 | ORF2110 | ORF2110-F: AGTGGACAATTGATTGGTGAA  ORF2110-R: CATCCATCCCTTACTTTGGAC | 597 |
| mPCR1 | ORF2819 | ORF2819-F: AGCAAAATGCCAAAACTCGT  ORF2819-R: CATCACTAAAGCCTCCCATTG | 471 |
| mPCR1 | *Imo*1118 | lmo1118-F: AGGGGTCTTAAATCCTGGAA  Imo1118-R: CGGCTTGTTCGGCATACTTA | 906 |
| mPCR1 | *Imo*0737 | lmo0737-F: AGGGCTTCAAGGACTTACCC  lmo0737-R: ACGATTTCTGCTTGCCATTC | 691 |
| mPCR1 | *Prs* | prs- F: GCTGAAGAGATTGCGAAAGAAG  prs-R: CAAAGAAACCTTGGATTTGCGG; | 370 |


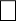


1

**FIGURE S1** PCR gel images obtained on 3% agarose gels to detect the serogroups

of *L. monocytogenes*

**Lane L:** 100bp DNA ladder; **Lanes 19,23,43:** *L. monocytogenes* serotypes

**Lanes 1-5, 6-18, 20-22, 24-41**: Untypable *Listeria* spp.

###

###

### **TABLE S2** Primers used for mPCR virulence profiling (Rawool *et al*., 2017)

| **Assay** | **Primer** | **Primer sequence (5’ 3’)** | **Product sizes (bp)** |
| --- | --- | --- | --- |
| mPCR1 | *InlB* | inlB-F: GATATTGTGCCACTTTCAGGTT  inlB-R: CCTCTTTCAGTGGTTGGGTT | 376 |
| mPCR1 | *PlcA* | plcA-F: CTGCTTGAGCGTTCATGTCTCATCCC  plcA-R: ATGGGTTTCACTCTCCTTCTAC | 1484 |
| mPCR1 | *HlyA* | hly-F: GTTAATGAACCTACAAGACCTTCC  hly-R: ACCGTTCTCCACCATTCCCA | 457 |
| mPCR1 | *ActA* | actA-F: TCGCCGCGGAAATTAAAAAAAGA  actA-R: ACGAAGGAACCGGGCTGCTAG | 839 |
| mPCR1 | *Iap* | iap-F: ACAAGCTGCACCTGTTGCAG  iap-R: TGACAGCGTGTGTAGTAGCA | 131 |
| mPCR2 | *InlA* | InlA-F: ACGAGTAACGGGACAAATGC  InlA-R: CCCGACAGTGGTGCTAGATT | 800 |
| mPCR2 | *InlC* | inlC-F: AATTCCCACAGGACACAACC  inlC-R: CGGGAATGCAATTTTTCACTA | 517 |
| mPCR2 | *InlJ* | inlJ-F: TGTAACCCCGCTTACACACAGTT  inlJ-R: AGCGGCTTGGCAGTCTAATA | 238 |

**FIGURE S2** PCR gel images obtained on 3% agarose gels to detect virulence

genes in *L. monocytogenes* isolates

**Lane L:** 100bp DNA ladder; **Lan**e **+**: positive control (ATCC 19111); **Lanes 1-**

**24:** *L. monocytogenes*
